# Massive Cerebral Infarction Following Facial Injection of Autologous Fat: A Case Report and Review of the Literature

**DOI:** 10.3389/fnhum.2021.610945

**Published:** 2021-02-09

**Authors:** Huan Qian, Yuxiao Ling, Mengwen Zhang, Cameron Lenahan, Chen Wang, Zhe Zheng, Anwen Shao, Jianmin Zhang

**Affiliations:** ^1^Department of Plastic Surgery, The Second Affiliated Hospital, School of Medicine, Zhejiang University, Hangzhou, China; ^2^School of Public Health, Hangzhou Medical College, Hangzhou, China; ^3^Center for Neuroscience Research, Loma Linda University School of Medicine, Loma Linda, CA, United States; ^4^Burrell College of Osteopathic Medicine, Las Cruces, NM, United States; ^5^Department of Neurosurgery, The Second Affiliated Hospital, School of Medicine, Zhejiang University, Hangzhou, China

**Keywords:** autologous fat grafting, vascular embolization, massive cerebral ischemia, hemiparesis, carotid artery

## Abstract

Facial fat grafting techniques often offer impressive surgical results. However, fatal complications, such as irreversible cerebral ischemia, blindness, and hemiplegia are associated with them. We have presented a case report of a patient who presented with a massive cerebral infarction, a serious complication of facial autologous fat injection. The patient was a 28-year-old female who experienced motor dysfunction of the left extremities, which was accompanied with loss of consciousness immediately following fat grafting for facial augmentation. Imaging studies suggested that the patient had a large cerebral infarction on the right frontal, temporal, and parietal lobes due to complete occlusion of her right external carotid artery. Emergency decompressive craniectomy was completed in addition to multiple follow-up medical treatments. The patient recovered after 4 months with reduced motor function in her left upper extremity. This report further summarizes published cases of massive cerebral ischemia after facial injection of autologous fat, as well as lists high-risk facial areas and critical warnings.

## Introduction

Massive cerebral infarction is a rare, but devastating complication that can occur after facial soft tissue augmentation. It can cause permanent blindness and hemiplegia (Egro and Coleman, 2020). We present a case of right massive cerebral infarction, which was secondary to autologous fat graft.

This report describes a 28-year-old female patient who presented with drowsiness and left limb motor weakness after a facial autologous fat graft. Neuroimaging studies revealed that the patient had a large cerebral infarction on the right frontal, temporal, and parietal lobes, as well as the basal ganglia. Computed tomographic angiography (CTA) of the neck suggested that the right external carotid artery was occluded.

Additionally, this report summarizes all similar published cases. The high-risk facial areas, initial clinical characteristics, treatment modalities, and prognosis of this serious complication after autologous fat facial injection were fully investigated.

## Case Presentation

**Figure 1:**
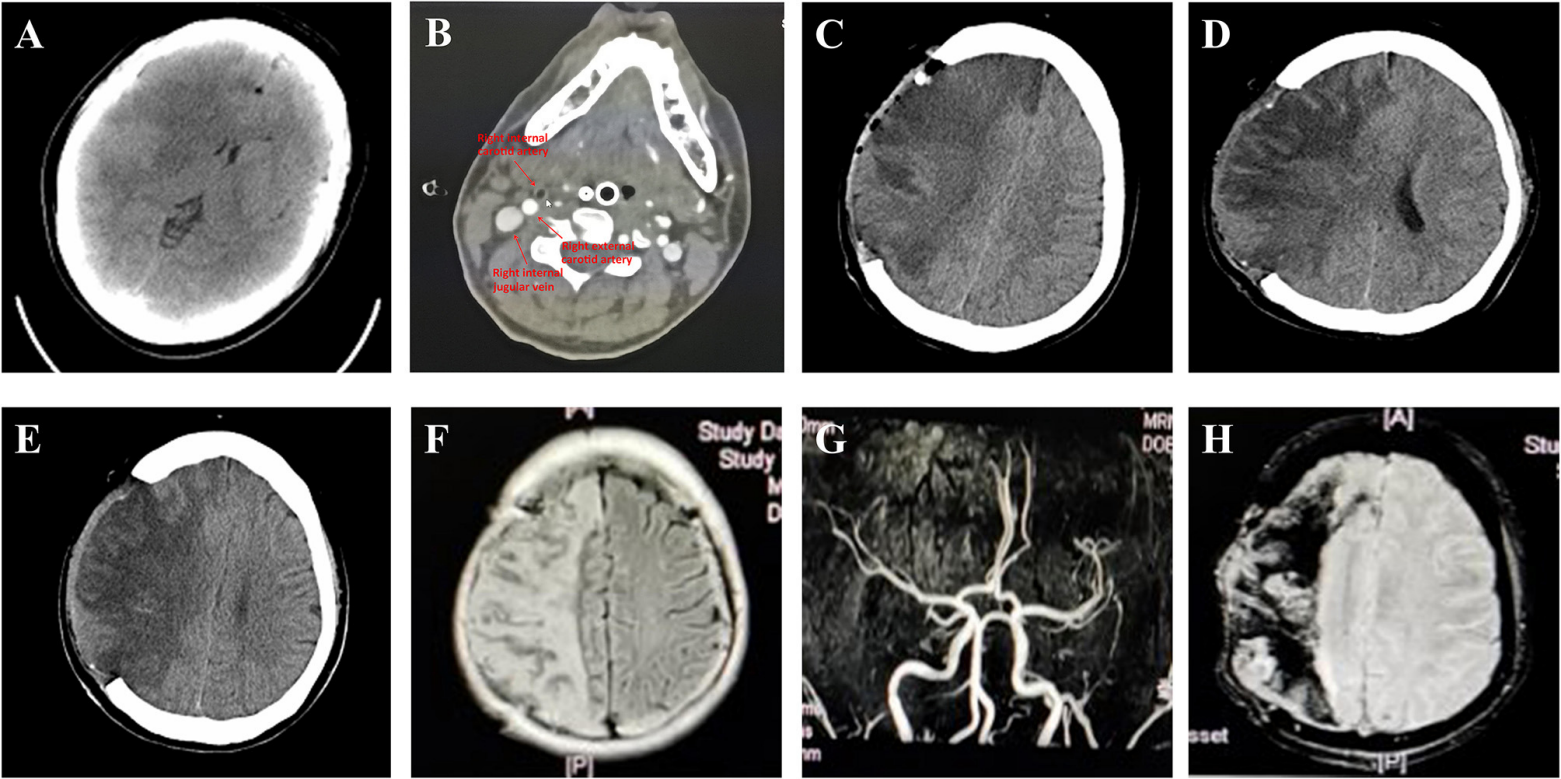
Imaging in different stages after cerebral infarction of this case. **(A)** Brain CT imaging of the patient shows massive cerebral infarction on the right frontal, temporal, and parietal lobes, and basal ganglia. **(B)** The neck CTA suggested that the right external carotid artery was occluded. ICA, ECA, or jugular vein are pointed out with arrows. **(C)** Post-surgery brain CT imaging of the patient. **(D)** Post-surgery Brain CT imaging of the patient 1 week post-surgery (2019-11-11). **(E)** Post-surgery Brain CT imaging of the patient 12 days post-surgery (2019-11-16). Images show improvement of edema, emorrhage absorbed. **(F)** The follow-up MRI-FLAIR performed 1 month after onset showed attenuated brain edema, and **(G)** MRA performed 2 months after onset showed vascular recanalization of intracranial area. **(H)** MRI-SWAN performed 2 months after onset showed the hemorrhage was gradually decreased with no other obvious hemorrhage.

A previously healthy 28-year-old woman underwent autologous fat grafting for facial augmentation by a plastic surgeon at a local clinic. A total volume of 77 ml of fat was grafted for the whole face under general anesthesia. Twenty ml fat was injected into each temple, 20 ml into the forehead, and 17 ml was injected in the cheeks. The patient experienced left limb movement disorder and drowsiness following the procedure. The patient was referred to the emergency room 24 h later with Glasgow Coma Scale (GCS) 14 points (Eye 4+Verbal 5+Motor 5). The left limb muscle strength was grade 2 (muscle can move only if the resistance of gravity is removed.), while the right limb muscle strength was grade 5 (muscle contracts normally against full resistance) at the time of admission. Emergency computed tomography (CT) suggested that the patient had a large cerebral infarction of the right frontal, temporal, and parietal lobes, as well as the basal ganglia, causing a midline shift to the left (Figure 1A). The neck CTA suggested that the right external carotid artery was occluded (Figure 1B). She underwent emergency decompressive craniectomy for decompression of right massive cerebral infarction. Because the arterial occlusion resulted from autologous fat embolization, it would not respond to traditional pharmacologic thrombolysis. The patient was transferred to the ICU after surgery, and was treated for dehydration, infection prophylaxis, gastric protection, and seizure prophylaxis treatment, etc.

The post-surgery CT scan revealed a large cerebral infarction on the right side, as well as hemorrhage and gas accumulation in the operative field (Figure 1C). Patient was treated for 10 days with intravenous mannitol injection. A subsequent CT examination, completed after 10 days of treatment mentioned above, confirmed a reduction in brain edema and hemorrhage (Figures 1D,E). Physical examination completed on day 11 revealed that the patient had a left limb muscle strength of 2, with numbness on the left limb, and no obvious abnormality on the right side. The patient was transferred to a rehabilitation hospital for further treatment.

The follow-up MRI-FLAIR (Figure 1F) showed attenuated brain edema, and MRA (Figure 1G) showed vascular recanalization of the intracranial area. An MRI-SWAN (Figure 1H) scan performed 2 months after onset showed that the hemorrhage was gradually decreased with no other obvious hemorrhage. The patient achieved better motor function of the left extremities (Her muscle strength recovered to grade 2+ on the left distal upper extremity, grade 4+ on the left proximal upper limb, and grade 5 on the left lower extremity) after 4 months of rehabilitation. Due to the outbreak of Corona Virus Disease-19 (COVID-19), the patient was discharged from the rehabilitation hospital in February. From that point forward, she continued her rehabilitation training from home. No imaging was performed on the patient at 4 months post-surgery. '

## Discussion

Autologous fat grafting is a widely performed procedure in facial surgery for soft-tissue correction, and was first published by Coleman in 1995 (Egro and Coleman, 2020). Autologous fat is an ideal filler for augmenting facial volume (Yoshimura and Coleman, 2015), during transplantation, fat aliquots, within 0.1 ml each pass, should be injected when the microcannula is withdrawn (Egro and Coleman, 2020). Common complications of this aesthetic surgery include volume under- or overcorrection, contour irregularities, prolonged bruising and swelling, infection, granulomas, and inflammation. However, severe complications, such as ocular embolization with visual loss, cerebral arterial infarct, and death are extremely rare (Yoshimura and Coleman, 2015; Cuzalina and Guerrero, 2018). Some complications may occur due to improper operations, therefore, one purpose of our case report is to remind the plastic surgeons paying more attention to every procedures, especially the injection operation.

We present a rare case of a female patient who had a severe cerebral infarction after autologous fat grafting, which resulted from right external carotid artery occlusion. In this case, we highly suspect that the fat tissue was injected into the right superficial temporal artery, and was then forced retrograde into the right external carotid artery (ECA). Given the demonstrated occlusion of the ECA, the fat emboli managed to travel retrograde from the right ECA to the carotid bifurcation and then anterograde into the right internal carotid artery. Finally, it caused right cerebral infarction in the right frontal, temporal, and parietal lobes, as well as basal ganglia. We then compared all the studies describing a massive stroke (excluding simple ocular artery embolization) after fat grafting (Table 1).

**Table 1:** Summary of facial autologous fat injection-induced cerebral ischemia.

**References**	**Age**	**Gender**	**Onset**	**Location**	**Volume per side**	**Artery**	**Onset symptoms**	**Evolution**
Huo et al. (2018)	33	F	0	Glabella	Not mentioned	LICA, LMCA	Right hemiparesis with loss of consciousness	Motor aphasia, reduce motor function on right limbs
	25	F	0	Glabella	Not mentioned	LMCA-M3, LACA	Right hemiparesis with loss of consciousness	Reduce motor function on right limbs
	24	F	2 h	Periocular	Not mentioned	LACA, RCCA, RICA, RMCA-M1, M3, RPCA-P2	Seizure, left hemiparesis and loss of consciousness	Died
	19	M	1 h	Glabella	Not mentioned	LMCA	Right hemiparesis with loss of consciousness	Aphasia, reduce motor function on right limbs
	28	M	5 h	Glabella	Not mentioned	Not examined	Seizure with loss of consciousness	Died
Wang et al. (2018)	22	F	0	Temporal	Not mentioned	RMCA	Left hemiparesis with loss of consciousness	Paralysis of the left limb, ageusia and vision loss in both eye
	30	F	0	Temporal	Not mentioned	Right brain hemisphere	Left limbs motor disturbance	Recovery of the left limb
Liu et al. (2018)	29	F	0	Left forehead	15 ml	LOA, LMCA	Left visual disturbance with no light sensation and right motor disturbance, nausea and vomit	Left eye blindness
	38	F	0	Left forehead	5 ml	LOA	Left visual disturbance and necrosis in the left forehead skin	Left eye blindness and skin scar
Kang et al. (2016)	32	F	30 min	Glabella	Not mentioned	LACA, LMCA, LOA	Right hemiparesis with loss of consciousness	Left eye blindness and able to walk and raise her arm with minimal resistance
Shen et al. (2016)	30	F	8 h	Temporal chin	Not mentioned	RECA, RICA	Left hemiparesis with loss of consciousness	Reduce motor function on right and left limbs
Thaunat et al. (2004)	39	M	0	Temporal Periocular glabella	17 ml	LACA, RACA, AcoA	Confusion, hypertonia, high blood pressure	Aphasia and paraplegia
Lee et al. (2011)	44	F	2 h	Periocular	Not mentioned	LOA, LMCA	Left visual loss, dysarthria and the skin necrosis	Left eye blindness and skin scar
Hong et al. (2014)	31	F	0	Glabella	Not mentioned	ROA, RMCA	Right visual disturbance and weakness in left arm	Decrease in visual acuity, the weakness in left arm improved to normal
Lee et al. (2012)	26	F	13 h	Face	Not mentioned	Not examined	Right visual loss and left hemiparesis	Not mentioned
Hu et al. (2011)	28	F	0	Temporal	Not mentioned	LMCA-M1	Drowsy, developed expressive aphasia, right hemiparesis	Considerable neurologic recovery (NIHSS score 6)
Yoon et al. (2003)	39	F	1 min	Glabella	5 ml	LICA	Drowsy, global aphasia, right hemiparesis	Died
Danesh-Meyer et al. (2001)	43	M	10 min	Nose lip Nasolabial groove	3 ml	LOA and MCA	Left visual loss and right hemiparesis	Left eye blindness
Wang et al. (2014)	22	F	5 h	Temporal forehead	25 ml/24 ml	LICA, LACA, LMCA	Right hemiparesis, aphasia, left visual loss	Left eye blindness, improvement in the aphasia and skin scar
Allali et al. (2006)	49	F	24 h	Glabella	Not mentioned	Multiple strips in LOA	Left visual loss	Left eye blindness
Feinendegen et al. (1998)	45	M	7 h	Nasolabial folds lower lip chin	Not mentioned	LMCA and ROA and choroidal arterioles	Aphasia and mild right sensorimotor hemiparesis	Not mentioned
Lee et al. (1996)	42	F	0	Nasolabial groove	0.5 ml	LOA	Left visual disturbance and drowsy	Decrease in visual acuity
Dreizen and Framm (1989)	44	F	0	Glabella	Not mentioned	ROA	Right visual loss and right hemicranial	Right eye blindness

*M, male; F, female; LMCA, left middle cerebral artery; LACA, left anterior cerebral artery; RMCA, right middle cerebral artery; RACA, right anterior cerebral artery; RCCA, right common carotid artery; RPCA, right posterior cerebral artery; RICA, right internal carotid artery; LICA, left internal carotid artery; RECA, right external carotid artery; LECA, left external carotid artery; LOA, left ophthalmic artery; ROA, right ophthalmic artery; AcoA, anterior communicating artery*.

### Demographic Information

General information of the patients included in the literature review is displayed, and of the 24 patients reported, 19 (79.17%) were women and 5 (20.83%) were men; the ages of the sample group ranged from 19 to 49 years (Mean = 32.9). Most of the patients were young and otherwise healthy women.

### Onset

In these cases, fat embolism after facial autologous fat injection developed either immediately after surgery or up to 3 days post-surgery. Patients who developed fat embolism experienced sudden headache, visual disturbance, cutaneous signs (e.g., pale skin), vomiting, and neurological symptoms (e.g., mental status changes, aphasia, or hemiparesis). Coma and death were also observed in some patients. As most cases of acute stroke with major artery embolisms occur within 24 h after plastic surgery, patients should be under professional medical observation for the first 24 h post-surgery.

### Location and Participating Arteries

The patient described in our case study had a very rare occurrence of fat embolism in the external carotid artery, which has not yet been reported. Embolic cerebral strokes after autologous fat injection are primarily seen in common carotid artery, internal carotid artery, anterior/middle/posterior cerebral artery, and the ophthalmic artery (Table 1). A retrograde intravascular fat emboli can be a significant contributor. In other cases, the intravascular fat travels retrograde through the communicating branches between the facial artery and the internal carotid artery, eventually leading to a fat embolism of major cerebral arteries or the ophthalmic artery. Potential blood routes include the dorsal, nasal, angular, supraorbital arteries, temporal superficial arteriovenous, or any anatomic variation. This leads to necrosis of the brain tissue and the optic nerve (Yoshimura and Coleman, 2015; Cuzalina and Guerrero, 2018; Sisti et al., 2019; Egro and Coleman, 2020).

**Figure 2:**
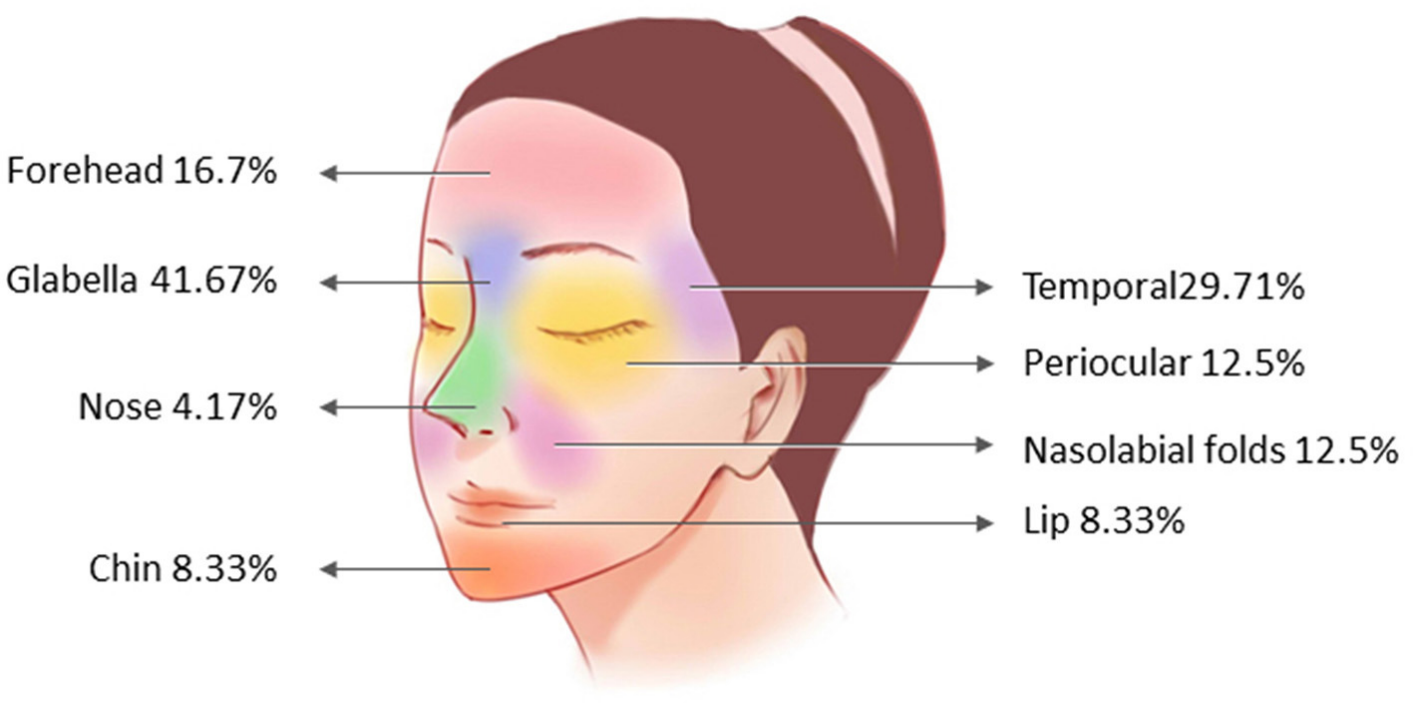
Distribution of fat receiving areas in patients with cerebral infarction.

The most dangerous injection areas (high-risk for fat embolism) were the glabella (angular and supraorbital arteries) (41.67%) and temporal area (temporal superficial arteriovenous) (29.17%). Areas with lower risk include the nose, lips, and chin. The artery deserving of special attention with a high risk of fat embolism is consistent with the corresponding area listed in Figure 2.

### Volume and Surgical Technique

Previous studies have demonstrated that injection of 0.5–30 ml in one area can lead to complications. Compared with breast or buttock augmentation, facial areas have a greater risk because small volumes of fat grafts can enter through the arteries, eventually flowing into the brain due to the rich vasculature. On the other hand, the physician may need to fill a moderate volume of fat in one area. Multiple dosages, number of injections, and massages may damage the local vascular network and increase the local pressure, which may increase the risk of arterial embolism.

A skilled surgical technique is essential to avoid post-surgical complications. Surgeons need to inject the fat slowly with low pressure, and they must ensure that there is no backflow of blood into the delivering syringe. Use of excessive force and velocity to administer injections may increase local pressure and the risk of retrograde travel of fat (Egro and Coleman, 2020). The cases described above provide evidence that minimal force should be exerted, and that a minimal dose should be used to seed the fat in tissue. Meanwhile, the use of a blunt cannula, withdrawn before injection, is also key in preventing intra-arterial injections.

### Outcome

Patients suffering from massive cerebral infarction in these cases received comprehensive treatment. The artery embolized with autologous fat particles did not respond to thrombolytic agents. However, there were several effective treatments, such as decompressive craniectomy paired with mannitol treatment, hyperbaric oxygen therapy, intravenous infusion of dextran glucose solution, hydrocortisone, anti-platelet agents, and systemic neurotrophic factor therapy. If detected early, vascular recanalization can be achieved via mechanical thrombectomy. However, currently, there are no published studies providing evidence of that. Partial recovery, aphasia, blindness, hemiparesis, and death were the prognoses seen in the patients analyzed in the literature review. Hence, the prevention of fat embolism is of utmost importance.

## Conclusions

To the best of our knowledge, this is the first paper summarizing the serious complications of fat transplantation in facial cosmetic surgery. Our understanding is that there might be a substantial number of unreported cases with similar complications of fat transplantation due to local legal issues. Therefore, it is of vital importance for surgeons to discern the causes of, as well as minimize, fatal complications.

## Data Availability Statement

The raw data supporting the conclusions of this article will be made available by the authors, without undue reservation.

## Ethics Statement

The studies involving human participants were reviewed and approved by the Human and Research Ethics committees of the Second Hospital of Zhejiang University. The patients/participants provided their written informed consent to participate in this study. Written informed consent was obtained from the individual(s) for the publication of any potentially identifiable images or data included in this article.

## Author Contributions

HQ, YL, and AS conceptualized the research project. HQ and YL drafted the manuscript. AS, ZZ, MZ, and CW reviewed and modified the manuscript. AS and JZ supervised the research and led the discussion. All authors approved the final version of the manuscript.

## Conflict of Interest

The authors declare that the research was conducted in the absence of any commercial or financial relationships that could be construed as a potential conflict of interest.
